# Single-Cell Mapping Reveals MIF-Centered Immunoregulatory Networks in Colorectal Cancer

**DOI:** 10.3390/ijms27031496

**Published:** 2026-02-03

**Authors:** Marios Gkoris, Ilias Georgakopoulos-Soares, Apostolos Zaravinos

**Affiliations:** 1 Department of Life Sciences, School of Sciences, European University Cyprus, Nicosia 1516, Cyprus; mg211332@students.euc.ac.cy (M.G.); ilias@austin.utexas.edu (I.G.-S.); 2 Cancer Genetics, Genomics and Systems Biology Laboratory, Basic and Translational Cancer Research Center (BTCRC), Nicosia 1516, Cyprus; 3 Division of Pharmacology and Toxicology, Dell Paediatric Research Institute, College of Pharmacy, The University of Texas at Austin, Austin, TX 78712, USA

**Keywords:** colorectal cancer, scRNA-seq, cell communication, tumor microenvironment

## Abstract

Colorectal cancer (CRC) progression is strongly shaped by the tumor microenvironment (TME), where complex interactions between epithelial, immune, and stromal cells orchestrate immune suppression and tumor evolution. To dissect these relationships at single-cell resolution, we analyzed CRC scRNA-seq datasets using Seurat for data integration and CellChat for ligand–receptor inference. We identified extensive cellular heterogeneity within the TME, dominated by CMS2/CMS3 epithelial states, SPP1^+^ tumor-associated macrophages, diverse T-cell subsets, and CXCR4^+^ B cells. Communication analysis revealed MIF-centered signaling—including MIF–CD74–CXCR4 and MIF–CD74–CD44—as the predominant axis linking tumor epithelial cells with T cells, B cells, and macrophage subpopulations. CMS3 epithelial cells displayed particularly strong connectivity to SPP1^+^ macrophages and cytotoxic lymphocytes through both MIF- and APP–CD74-mediated pathways. Differential gene expression confirmed elevated levels of MIF, CD74, CD44, and SPP1 in tumor tissues, while pathway enrichment analyses highlighted cytokine signaling, antigen presentation, and chemokine-regulated immune modulation as key biological processes. Collectively, our study provides a high-resolution map of CRC intercellular communication and identifies MIF-CD74-associated signaling as a central immunoregulatory hub with potential relevance for therapeutic targeting and biomarker development.

## 1. Introduction

Colorectal cancer (CRC) is the third most diagnosed malignancy worldwide and a major contributor to cancer-related mortality [1,2]. Its initiation and progression are not solely determined by genetic alterations within tumor cells but are profoundly shaped by the tumor microenvironment (TME)—a heterogeneous ecosystem composed of immune cells, stromal components, endothelial cells, and diverse epithelial subpopulations [3,4]. The dynamic interactions occurring within the TME regulate processes such as immune evasion, chronic inflammation, metabolic rewiring, and metastatic behavior [5]. Despite significant advances in understanding CRC biology, the mechanisms governing intercellular crosstalk between malignant cells and their surrounding immune cell populations remain only partially resolved.

Advances in single-cell RNA sequencing (scRNA-seq) have transformed our ability to dissect the cellular heterogeneity of human tumors [6]. By profiling transcriptomes at single-cell resolution, scRNA-seq enables the identification of tumor dynamics and heterogeneity, rare cell populations, cellular states, lineage trajectories, and communication networks that are otherwise undetectable in bulk analyses [7,8,9]. In CRC specifically, scRNA-seq studies have revealed key functional immune subsets—including exhausted CD8^+^ T cells, SPP1^+^ tumor-associated macrophages (TAMs), and regulatory B cells [10,11,12], as well as epithelial diversity associated with the Consensus Molecular Subtypes (CMS1–CMS4) [13]. However, the ligand–receptor signaling interactions that mediate communication between CMS-specific tumor cell states and immune cells remain insufficiently characterized.

Computational frameworks such as CellChat [14,15], CellPhoneDB [16,17], and NicheNet [18] have further enabled systematic inference of intercellular communication networks by integrating curated ligand–receptor databases with single-cell transcriptional data. These tools provide insight into immunoregulatory interactions governing tumor progression, including macrophage-derived SPP1–CD44 signaling, dendritic cell inhibitory pathways (e.g., CD24-Siglec10 axis), and cytokine-mediated suppression of cytotoxic T cell activity [19,20]. Yet, comparative analyses across CRC epithelial subtypes and their immune interfaces remain limited, especially concerning Macrophage Migration Inhibitory Factor (MIF)-centered signaling, the Amyloid Precursor Protein (APP)–Cluster of Differentiation 74 (CD74) axis, and their involvement in shaping the immunosuppressive milieu.

The MIF–CD74 axis regulates the tumor–immune interactions in CRC. MIF produced by tumor and immune cells signals through CD74/CD44 to activate the ERK1/2, PI3K–AKT, NF-κB, and β-catenin pathways, promoting tumor growth, immune evasion, and poor prognosis [21,22,23,24,25,26,27,28] (Figure S1). Given its broad activity across immune cell types, the MIF–CD74 axis is emerging as a druggable target, with inhibitors and neutralizing antibodies showing potential to restore antitumor immunity and enhance T cell function in preclinical models [29].

Here, we leverage CRC scRNA-seq datasets [8,10] to delineate cellular composition, epithelial heterogeneity, and immune infiltration within the TME, focusing on dominant CMS2/3 epithelial populations, SPP1^+^ macrophages, and lymphocyte subsets. Beyond confirming MIF–CD74 signaling in CRC, we delineate three CRC-specific insights: (i) multi-lineage engagement of MIF signaling that prominently recruits CD19^+^CD20^+^ B cells via CD74 and CXCR4; (ii) a CMS3-preferential APP–CD74 axis linking metabolically rewired tumors to B cells and SPP1^+^ macrophages; and (iii) subtype-specific communication strength, with CMS3 showing denser edges to SPP1^+^ TAMs and CD8^+^ T cells than CMS2. These refinements position MIF-centered signaling as a multi-node immunoregulatory hub in CRC.

Overall, our work aims to provide an integrated, single-cell-level perspective of the interactions between CRC epithelial cells and the immune ecosystem, thereby uncovering novel communication routes that may guide biomarker development and immunotherapeutic strategies.

## 2. Results

### 2.1. Cellular Architecture of CRC and Normal Mucosa

To characterize the cellular architecture of CRC at single-cell resolution, we first integrated and normalized the scRNA-seq datasets and performed PCA followed by UMAP. This workflow revealed clear separation of major cellular compartments, including immune, stromal and epithelial lineages (Figure S2).

**Figure 1:**
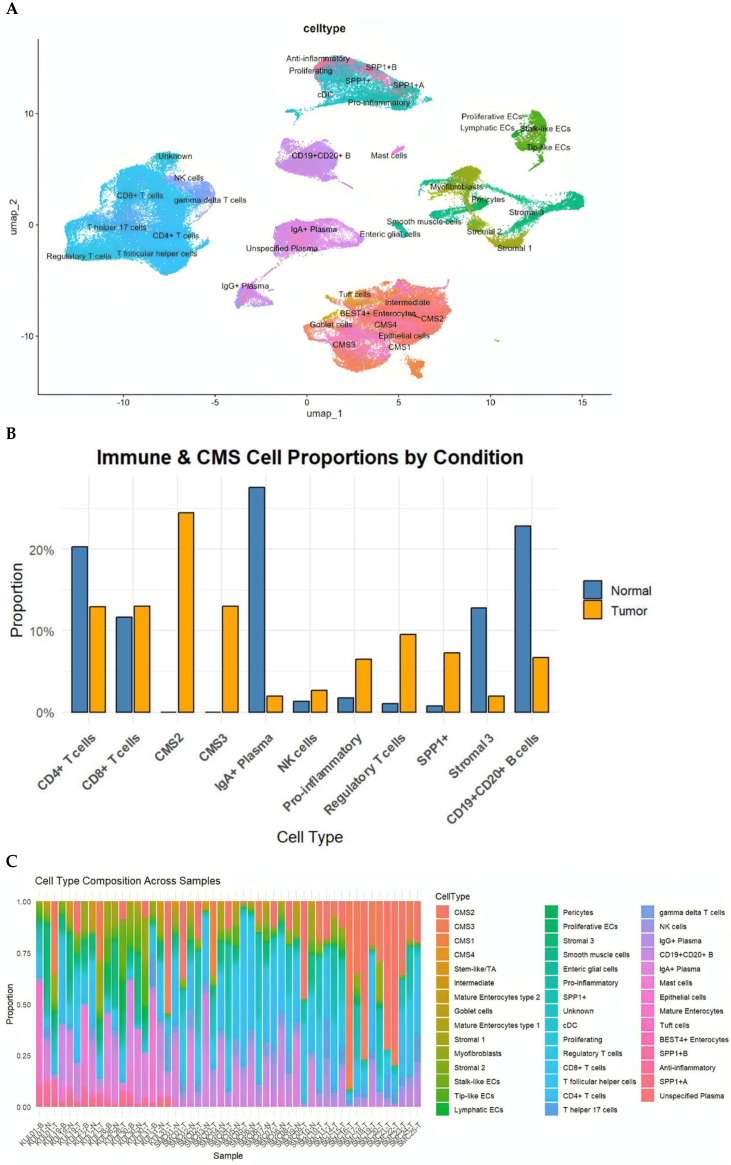
(**A**) UMAP visualization of the integrated dataset, annotated into epithelial, stromal, immune, and specialized cell populations based on canonical marker genes. Subpopulations include CD4^+^ and CD8^+^ T cells, Tregs, Th17, Tfh, B cells, IgA^+^/IgG^+^ plasma cells, dendritic cells, NK cells, mast cells, multiple stromal lineages, and epithelial cell states including CMS1–CMS4, goblet cells, tuft cells, and BEST4^+^ enterocytes. (**B**) Total pro-portions of major cell classes in normal versus cancer samples, highlighting expansion of CMS2/3 tumor epithelial cells, pro-inflammatory macrophages, NK cells, Tregs, and SPP1^+^ macrophages in tumors. (**C**) Distribution of the eleven most abundant cell types in cancer and normal tissues, illustrating shifts in epithelial, immune, and stromal populations.

Within the immune compartment, we identified diverse T-cell subsets (CD4^+^, CD8^+^, Tregs, Th17, Tfh), B cells and plasma cells (IgA^+^ and IgG^+^), macrophages (pro-inflammatory, anti-inflammatory, and SPP1^+^ subsets), dendritic cells, mast cells, and NK cells. Stromal populations included fibroblasts, myofibroblasts, perivascular cells, smooth muscle cells, and enteric glial cells. Epithelial cells were annotated according to the Consensus Molecular Subtypes (CMS1–CMS4) and further included BEST4^+^ enterocytes, goblet cells, tuft cells and intermediate enterocyte states (Figure 1A).

This comprehensive annotation underscores the pronounced cellular heterogeneity of the CRC TME and provides the foundation for downstream intercellular communication analysis.

We next compared the cellular compositions of CRC tumors and matched normal mucosa. Tumor tissues demonstrated a significant expansion of epithelial tumor cells of CMS2 (canonical) and CMS3 (metabolic) subtypes, alongside increased frequencies of NK cells, pro-inflammatory macrophages, Tregs, and particularly SPP1^+^ macrophages, a hallmark of immunosuppressive TMEs. In contrast, normal tissues were enriched for mature absorptive enterocytes, goblet cells, endothelial cells, and multiple stromal subpopulations (Figure 1B). Although all CMS subtypes were represented in the datasets, CMS2 and CMS3 epithelial states predominated in the integrated analysis, consistent with their higher prevalence in CRC cohorts. CMS1 and CMS4 cells were detected at lower frequencies

Analysis at the individual-sample level further revealed substantial inter-tumoral heterogeneity, with some tumors dominated by CMS2/CMS3 epithelial populations and others characterized by abundant inflammatory macrophages or endothelial cells (Figure 1C). These differences emphasize that CRC tumors exhibit unique cellular ecosystems that are likely to influence intercellular signaling programs.

### 2.2. Global Intercellular Communication Networks in CRC

**Figure 2:**
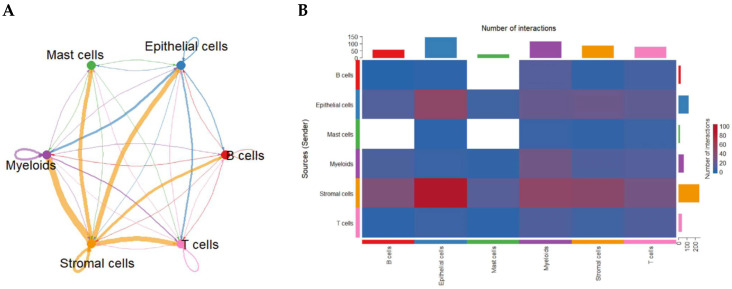
Global intercellular communication network in the CRC tumor microenvironment inferred by CellChat. (**A**) Circular communication network displaying the strength of predicted ligand–receptor interactions among major cell groups. Line thickness represents overall communication probability; circular edges indicate autocrine signaling. (**B**) Heatmap quantifying the number of predicted ligand–receptor pairs between sender (rows) and receiver (columns) cell types. Stromal and myeloid populations exhibit the highest outgoing and incoming communication frequencies.

To uncover how cell populations interact within the CRC TME, we applied CellChat to infer ligand–receptor-based communication networks. Global analysis revealed that stromal cells function as dominant signaling hubs, exhibiting the strongest outgoing and incoming communication interactions with nearly all other cell types. Myeloid cells—particularly macrophages—also demonstrated high bidirectional signaling strength. Autocrine loops were prominent in stromal and myeloid compartments, indicating reinforcement of their intrinsic signaling programs. In contrast, B cells and mast cells displayed comparatively weaker communication profiles (Figure 2A).

An interaction heatmap corroborated these findings, showing extensive stromal-to-epithelial, stromal-to-myeloid, and myeloid-to-T-cell signaling (Figure 2B), highlighting the coordinated network supporting CRC progression.

### 2.3. Communication Between CMS2/CMS3 Cancer Epithelial Subtypes and Immune Populations

We subsequently focused on CMS2/CMS3-specific interactions, which constitute the major epithelial states. Relative to normal mucosa, tumor samples exhibited stronger stromal → immune and myeloid → T-cell signaling, consistent with an inflammatory and immunosuppressive milieu. MIF-centered axes (MIF–CD74–CXCR4/CD44) were predominantly detected in tumor tissues, linking CMS2/CMS3 epithelial states to B cells, CD8^+^ T cells, and SPP1^+^ macrophages. In contrast, normal tissues showed comparatively homeostatic epithelial–stromal signaling with reduced MIF-axis engagement.

**Figure 3:**
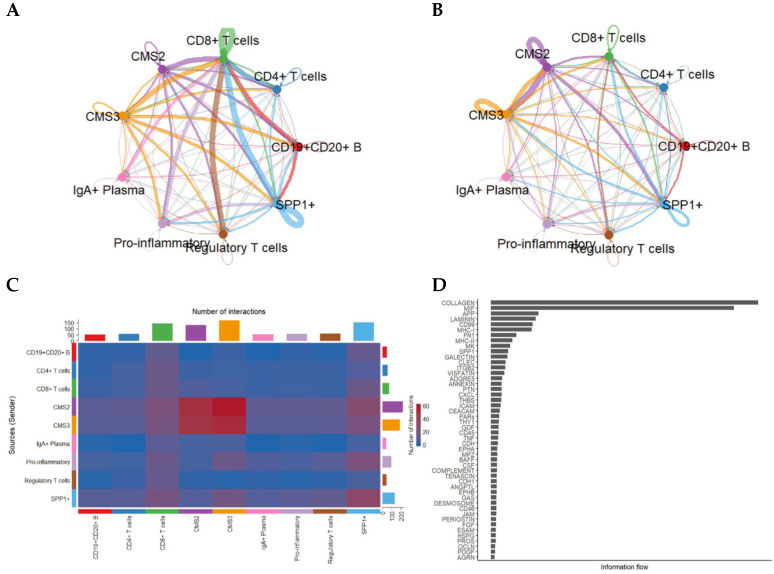
Intercellular communication patterns stratified by CMS2 and CMS3 tumor epithelial subtypes. (**A**) Network visualization showing the number of predicted interactions between CMS2/CMS3 epithelial cells and immune/stromal subsets. (**B**) Corresponding network based on total communication strength (information flow). (**C**) Heatmap showing pairwise interaction counts across epithelial and immune populations. (**D**) Ranking of signaling pathways based on total information flow, illustrating predominant pathways enriched in CMS2/CMS3-mediated communication.

Because epithelial states strongly influence tumor–immune interactions, we next resolved communication patterns by separating tumor cells into CMS2 and CMS3 subtypes. This refinement revealed striking differences in how each epithelial subtype interacts with the immune milieu (Figure 3).

CMS3 cells, characterized by metabolic reprogramming, displayed intense signaling with CD8^+^ T cells, SPP1^+^ macrophages, and regulatory T cells, whereas CMS2 cells exhibited broader interactions with both adaptive and innate immune cells.

Dense communication edges—particularly involving CMS3 → SPP1^+^ and CMS3 ↔ CD8^+^ T cells—suggest that metabolic epithelial states may play specialized roles in shaping immune suppression and cytotoxic T-cell dysfunction.

### 2.4. Key Ligand–Receptor Pathways Driving Tumor–Immune Crosstalk

**Figure 4:**
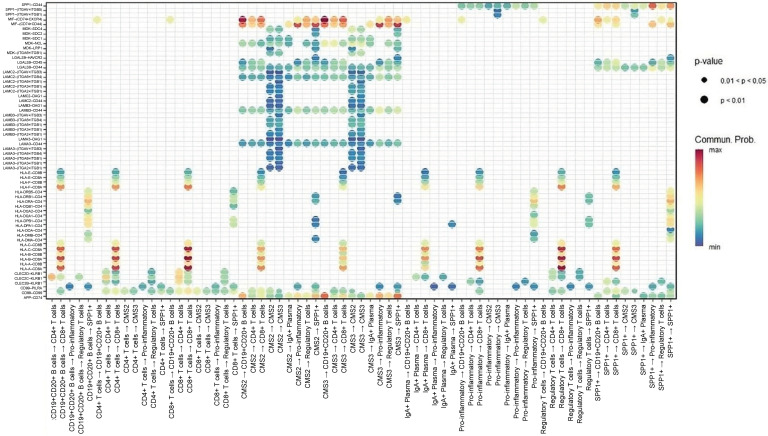
Dot-plot representation of key ligand–receptor interactions between epithelial and immune populations. Dot size reflects statistical significance (*p*-value) and color intensity indicates communication probability. Highlighted interactions include MIF–(CD74 + CXCR4) and MIF–(CD74 + CD44) linking CMS2/3 epithelial cells with CD4^+^/CD8^+^ T cells, B cells, pro-inflammatory macrophages, and SPP1^+^ macrophages, as well as APP–CD74 communication between CMS3 cells and B-cell/macrophage lineages.

Focused ligand–receptor analysis highlighted several central immunomodulatory axes mediating communication between cancer cells and immune subsets (Figure 4). First, MIF–(CD74 + CXCR4) is a dominant axis across CMS2 and CMS3 cells, transmitting signals to CD4^+^/CD8^+^ T cells, B cells, pro-inflammatory macrophages, and SPP1^+^ macrophages. In addition, the MIF–(CD74 + CD44) axis was particularly enriched in interactions with macrophages, suggesting cooperative inflammatory and immunosuppressive signaling. Furthermore, the APP–CD74 axis is primarily linking CMS3 epithelial cells to B cells and SPP1^+^ macrophages, indicating a specialized role in metabolic tumors. Finally, the HLA-A–CD8A/CD8B axis is reflecting potential MHC-I-mediated activation of cytotoxic T cells, though likely subdued by concurrent immunosuppressive axes. These data collectively indicate that MIF-centered signaling pathways form the core of CRC communication networks.

### 2.5. Differential Gene Expression Confirms Predicted Signaling Axes

**Figure 5:**
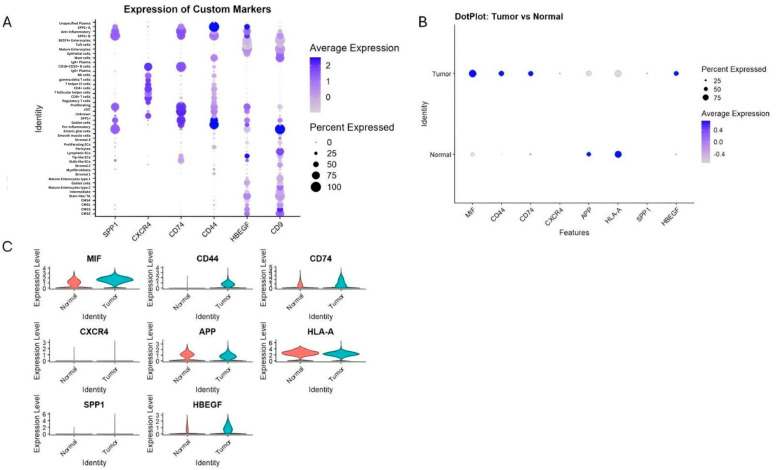
Expression of key ligand and receptor genes involved in predicted communication pathways. (**A**) Dot plot of mean expression and percentage of expressing cells across all cell types for SPP1, CXCR4, CD74, CD44, HBEGF, and CD9. (**B**,**C**) Comparison of expression levels of MIF, CD44, CD74, SPP1, CXCR4, APP, HLA-A, HBEGF, and CD9 between cancerous and normal tissues, revealing tumor-specific upregulation of immunoregulatory molecules and macrophage markers.

Transcriptomic comparisons were performed on GSE132465 and validated using GSE144735 and bulk datasets (GSE41258, GSE90627, GSE117606). Expression analysis of key genes involved in inferred communication pathways revealed various cell type-specific patterns (Figure 5). For example, we found that CD44 and CD74 were highly expressed in SPP1^+^ and pro-inflammatory macrophages. These expression levels refer to total transcript levels, since splice variant resolution is not feasible with current scRNA-seq data. Additionally, CXCR4 was predominantly restricted to B cells, consistent with B-cell participation in MIF–CXCR4 signaling. Furthermore, MIF showed widespread expression among tumor epithelial cells and myeloid populations. Also, HBEGF and CD9 were enriched in CMS3 epithelial cells, fitting their metabolic phenotype. Finally, APP and HLA-A exhibited higher baseline expression in normal tissues.

These findings validate CellChat-predicted interactions and highlight functional compartmentalization of ligand–receptor signaling in the CRC TME.

### 2.6. Pathway Enrichment Reveals Immunoregulatory and Metabolic Signatures

**Figure 6:**
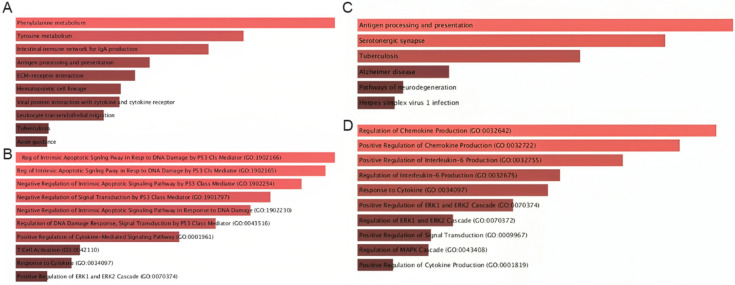
Pathway enrichment analysis of genes participating in MIF-centered signaling axes. (**A**) KEGG pathways enriched among genes in the MIF–CD74–CXCR4–CD44 axis, highlighting associations with cytokine–cytokine receptor interactions, antigen presentation, leukocyte migration, and inflammatory responses. (**B**) GO Biological Process terms for MIF-centered signaling indicating enrichment in immune activation, cytokine regulation, apoptotic pathways, and p53-related signaling. (**C**) KEGG pathways enriched for APP-CD74-related genes, including antigen presentation, ERK/MAPK activation, and viral infection pathways. (**D**) GO Biological Process terms emphasizing cytokine production, chemokine regulation, and MAPK-driven activation cascades.

We then evaluated the pathways associated with the top communication axes using Enrichr. The MIF–CD74–CXCR4–CD44 axis showed particularly diverse enrichment patterns. As illustrated in Figure 6A, this axis was linked not only to canonical immune-related pathways—such as antigen processing and presentation, intestinal immune networks for IgA production, hematopoietic lineage differentiation, and leukocyte transendothelial migration—but also to broader metabolic and stress-response pathways, including phenylalanine and tyrosine metabolism, and ECM–receptor interactions (Figure 6A). In addition, the MIF–CD74–CXCR4–CD44 axis exhibited a strong enrichment of p53-mediated intrinsic apoptotic signaling, regulation of DNA-damage responses, cytokine-mediated signaling, and T-cell activation, underscoring the dual role of this communication route in both immune orchestration and tumor-suppressive/apoptotic programs (Figure 6B).

Similarly, the APP–CD74 axis was enriched for pathways that converge on antigen presentation and inflammatory signaling (Figure 6C). In particular, we found high enrichment in pathways such as antigen processing and presentation, serotonergic synapse, tuberculosis-related immune activation, and neuroinflammatory and neurodegeneration-associated processes, suggesting a conserved stress-response component. In parallel, the APP–CD74 axis was mainly enriched for the positive regulation of chemokine and IL-6 production, MAPK/ERK cascade activation, and cytokine-responsive pathways, indicating that APP–CD74 interactions are tightly coupled to pro-inflammatory and MAPK-driven signaling circuits (Figure 6D).

Together, these enriched pathways reveal that both communication axes converge on key biological processes such as inflammation, antigen presentation, cytokine signaling, apoptosis control, and MAPK/ERK-mediated stress responses. Their combined influence highlights a multidimensional impact on CRC biology, affecting tumor–immune interactions, immune evasion mechanisms, stress and apoptotic responses, and metabolic remodeling. These findings support the hypothesis that MIF–CD74–CXCR4–CD44 and APP–CD74 represent central communication hubs that integrate immune regulation with tumor cell survival and adaptive signaling programs in colorectal cancer.

## 3. Discussion

The CRC TME is highly dynamic and different cell types including epithelial, stromal, and immune cells continuously shape one another’s phenotypes through ligand–receptor interactions. Although previous studies have mapped transcriptional heterogeneity within CRC [30,31,32,33], the functional communication architecture governing tumor–immune crosstalk has remained under-characterized. Our integrative scRNA-seq and CellChat analysis provides what is, to our knowledge, one of the most detailed intercellular signaling maps for CRC, revealing extensive networks dominated by epithelial (CMS2/3), myeloid, and stromal populations.

Our findings confirm and extend prior evidence that the CRC TME is enriched for SPP1^+^ TAMs and dysfunctional T-cell subsets, particularly exhausted CD8^+^ T cells and regulatory T cells [34,35]. SPP1^+^ TAMs have increasingly been implicated in immune suppression, angiogenesis, matrix remodeling, and poor prognosis [36,37,38,39], and our results highlight that their immunosuppressive activity is not isolated, but embedded in a broader network of reciprocal signaling, especially via MIF-centered pathways. Importantly, we show that CMS3 epithelial cells—characterized by metabolic rewiring—engage SPP1^+^ macrophages more intensely than CMS2 cells, suggesting subtype-specific immunoregulatory circuits that may influence patient stratification and therapy response.

A key contribution of our work is the characterization of MIF–CD74–CXCR4 and MIF–CD74–CD44 axes as central hubs of CRC communication, extending previous findings. While MIF has been widely recognized as a pro-tumor cytokine [40], earlier single-cell datasets have not comprehensively mapped its multi-lineage engagement across epithelial, T-cell, B-cell, and macrophage populations in CRC. We demonstrate that CMS2/3 cancer cells serve as major MIF sources, while macrophages, T cells, and B cells act as dominant recipients. This multi-axis MIF signaling is biologically meaningful. CD74 is known to stabilize MIF binding, CD44 functions as a co-receptor that amplifies MAPK/NF-κB/AKT signaling, and CXCR4 recruitment enhances chemotaxis and motility [41]. Together, these interactions form a paracrine architecture capable of simultaneously promoting proliferation, immune cell dysfunction, and inflammatory remodeling of the TME. Our analysis therefore refines prior models of MIF biology by showing that CRC leverages MIF signaling not through a single pathway but through a multi-node, cell-type-specific communication web.

Another novel insight is the active participation of B cells in CRC signaling networks. Historically, B cells were thought to play ambiguous or context-dependent roles in solid tumors, with conflicting evidence regarding whether they support or suppress antitumor immunity [42,43]. Recent work in melanoma and hepatocellular carcinoma has implicated B cells in tertiary lymphoid structure formation and antigen presentation [44,45]. However, CRC-focused scRNA-seq studies have not emphasized B-cell signaling. Our dataset reveals that CD19^+^CD20^+^ B cells show high expression of CD74 and CXCR4, making them major recipients of MIF-based signaling. This suggests that B cells may be drawn into immunoregulatory circuits driven by tumor epithelial cells and myeloid subsets. Given that B-cell-targeted therapies already exist in the clinic, our findings raise the intriguing possibility that immune-regulatory B cells could represent an underappreciated therapeutic target or biomarker in CRC.

In addition to MIF, we identified APP–CD74 as another important communication axis, particularly in CMS3 tumors. Although APP is generally studied in neuronal contexts, accumulating evidence links APP to epithelial proliferation, ERK/MAPK activation, and inflammatory signaling [46,47]. In our analysis, APP-mediated signaling connects CMS3 epithelial cells with B cells and macrophages, suggesting that metabolic tumors may use APP–CD74 to reinforce immunosuppressive circuits. This adds a previously unrecognized dimension to CRC ligand–receptor biology and may be particularly relevant to tumors that exhibit metabolic rewiring, lipid signaling alterations, or enhanced ERK/MAPK activation.

Differential gene expression patterns support these communication networks. Tumor tissues showed marked upregulation of MIF, CD44, CD74, SPP1 and HBEGF, all known drivers of pro-tumor inflammation, macrophage recruitment, and epithelial plasticity [48]. Conversely, normal tissues showed higher expression of APP and HLA-A, which may reflect baseline antigen-presentation capacity and epithelial homeostasis. The simultaneous elevation of CD44 and SPP1 in macrophages—together with their connectivity to CMS3 epithelial cells—suggests a feed-forward immunosuppressive circuit wherein tumor epithelial cells activate macrophages that, in turn, reinforce epithelial survival and immune evasion.

Pathway enrichment further strengthens these biological interpretations, revealing that MIF- and APP-associated genes are enriched in cytokine–cytokine receptor interactions, antigen processing and presentation, chemokine signaling, p53-mediated apoptosis, and leukocyte migration. These pathways are central to T-cell exhaustion, stromal remodeling, and resistance to immunotherapy. Importantly, the involvement of ERK/MAPK, NF-κB, and AKT signaling aligns with known molecular vulnerabilities of CRC and corroborates emerging therapeutic interest in targeting MIF or CD74. Indeed, several preclinical studies have shown that MIF blockade or CD74 inhibition can reduce tumor burden, reprogram TAMs, and restore T-cell cytotoxicity [25,49], supporting the translational relevance of our findings.

Overall, the novel contributions of our analysis are as follows: (i) We identify CD19^+^CD20^+^ B cells as prominent recipients (high CD74/CXCR4) of tumor derived MIF signaling, positioning B cells within the immunoregulatory circuit rather than peripheral bystanders. This multi lineage engagement (epithelial → B cells/macrophages/T cells) was not mapped as a multi node network in the original studies. (ii) We delineate an APP–CD74 axis most pronounced in CMS3 epithelial states, linking metabolic tumors to B cells and SPP1^+^ TAMs—an interaction not highlighted in the original reports. (iii) We show denser CMS3 ↔ SPP1^+^ TAM and CMS3 ↔ CD8^+^ T communication edges compared to CMS2, refining how metabolic rewiring may align with immune suppression circuitry.

Despite these strengths, some limitations remain. Computational inference cannot fully capture receptor–ligand affinity, spatial constraints, or the impact of protein-level modifications. Furthermore, scRNA-seq data inherently underrepresent low-abundance cytokines and receptors. A specific limitation is the inability to distinguish between CD44 splice variants using standard scRNA-seq data; our analysis reflects total CD44 gene expression rather than variant-specific isoforms such as the metastasis-associated CD44v6, which may play distinct functional roles in tumor progression. Our CellChat analysis focused on tumor samples to characterize immunoregulatory networks within the TME rather than comparing tumor versus normal communication patterns. Our analysis relies heavily on high-quality annotations and available curated ligand–receptor databases, which may not include all CRC-relevant interactions. Future integration of spatial transcriptomics, multiplex imaging, and functional perturbation assays—especially involving B cells, MIF and APP—will be essential for mechanistic validation. Additionally, full-length scRNA-seq or targeted validation approaches would be valuable to resolve CD44 variant-specific expression patterns in the CRC microenvironment. Future studies with larger normal tissue cohorts could examine how communication networks evolve during CRC tumorigenesis. Stratifying communication patterns by microsatellite instability (MSI) status, KRAS/BRAF genotype, and therapeutic regimen could refine the clinical implications of our findings.

Overall, our study provides a high-resolution atlas of CRC intercellular signaling, identifying MIF-centered pathways and APP–CD74 interactions as dominant immunoregulatory circuits that connect epithelial tumor states with myeloid, lymphoid and stromal compartments. These findings shed new light on the complex orchestration of tumor–immune interactions in CRC and highlight several promising targets for therapeutic intervention, including CD74, CXCR4 and MIF. Future validation and clinical correlation may pave the way for leveraging these axes in immunotherapy, macrophage-reprogramming strategies and subtype-specific biomarker development.

## 4. Materials and Methods

### 4.1. Datasets and Study Design

Single-cell RNA sequencing (scRNA-seq) data were obtained from the Gene Expression Omnibus (GEO) under accession numbers GSE132465 (https://www.ncbi.nlm.nih.gov/geo/query/acc.cgi?acc=GSE132465; accessed on 15 August 2025) and GSE144735 (https://www.ncbi.nlm.nih.gov/geo/query/acc.cgi?acc=GSE144735; accessed on 15 August 2025) [10]. GSE132465 served as the primary dataset, comprising 63,689 cells from 23 colorectal cancer patients, including 23 primary tumor samples and 10 matched normal mucosa samples. Dataset GSE144735 included 27,414 cells from 6 patients. Notably, this dataset sampled both tumor core and tumor border regions alongside matched normal tissue, providing 3 samples per patient (18 total samples from 6 patients). Combined, the integrated dataset comprised 51 individual tissue samples (29 tumor core, 6 tumor border, 16 normal) from 29 unique patients, enabling comprehensive assessment of cellular heterogeneity across spatial and inter-patient contexts. No subtype pre-selection was applied; cell states were annotated post hoc based on CMS marker signatures.

Because both datasets provided UMI count matrices and pre-annotated cell identities, no raw FASTQ preprocessing was required.

### 4.2. Quality Control and Preprocessing

All preprocessing was conducted in R using Seurat v4.3.0 [50,51]. Cells were filtered using the following criteria: mitochondrial gene percentage < 15%, erythrocyte gene percentage < 3%, at least 500 UMIs, and >200 detected genes per cell. Data was normalized using Seurat’s *LogNormalize* method to ensure comparability across patients and sequencing batches.

### 4.3. Dataset Integration and Dimensionality Reduction

To minimize batch effects between datasets, we used Seurat’s *FindIntegrationAnchors* and *IntegrateData* functions (https://satijalab.org/seurat/articles/integration_rpca.html; accessed on 15 August 2025) [50]. Integration anchors were computed based on shared highly variable genes across datasets. The integrated object was subjected to PCA for dimensionality reduction, and UMAP for visualization of global cell-state architecture. Major cell types were annotated using the provided labels from the original studies, supplemented by canonical marker genes where needed.

### 4.4. Cell Type Annotation

Immune, stromal and epithelial populations were labeled using established marker panels, enabling identification of T-cell subsets (CD4^+^, CD8^+^, Tregs, Th17, Tfh), B cells and plasma cells (CD19^+^CD20^+^, IgA^+^, IgG^+^), macrophage states (pro-inflammatory, anti-inflammatory, SPP1^+^), dendritic cells, mast cells, NK cells, stromal populations (fibroblasts, myofibroblasts, perivascular cells, endothelial subsets, glial cells), epithelial clusters, including CMS1–CMS4, BEST4^+^ enterocytes, intermediate enterocytes, tuft cells, and goblet cells [51,52,53,54].

### 4.5. Differential Gene Expression Analysis

Differential gene expression between tumor and normal samples was performed using a pseudobulk approach with the *limma* package. UMI counts were first aggregated across cells within each cell type and patient sample using Seurat’s *AggregateExpression* function. The resulting pseudobulk count matrices were then analyzed using limma-voom, which applies variance modeling and empirical Bayes moderation for stable statistical estimates. Differential expression was tested using moderated *t*-tests with Benjamini–Hochberg false discovery rate (FDR) correction (FDR < 0.05). This pseudo-bulk approach provides superior control of false discovery rates and better accounts for patient-level biological variability compared to cell-level tests [55,56].

### 4.6. Intercellular Communication Analysis

Intercellular signaling networks were inferred using CellChat [15]. A CellChat object was constructed from the integrated scRNA-seq dataset, using cell type labels as group identifiers. The CellChatDB.human ligand–receptor database was used to predict valid interactions [57]. CellChat performed preprocessing and probability inference of ligand–receptor interactions, quantification of interaction number and interaction strength, identification of dominant signaling pathways, visualization through circular networks, heatmaps, and information-flow barplots. Due to computational limitations, CellChat analyses were performed primarily on GSE132465, which provided the largest cell count and statistical depth.

### 4.7. Pathway Enrichment Analysis

Genes involved in major predicted signaling axes—particularly MIF–CD74–CXCR4–CD44 and APP–CD74—were analyzed using Enrichr [58,59]. Enrichment was performed across KEGG pathways and Gene Ontology (GO) Biological Process terms. Enrichment results were visualized through barplots and ranking metrics to identify key immune and metabolic pathways associated with inferred communication networks.

### 4.8. External Validation Datasets

To validate the expression and prognostic relevance of communication-related genes, external CRC datasets and the IMvigor210 checkpoint-therapy cohort were included [59,60,61]. These datasets were used to compare expression patterns, examine pathway activation consistency, and assess associations with survival or immunotherapy responsiveness.

### 4.9. Statistical Analysis

All statistical analyses were performed in R (v4.2+). Unless otherwise stated, differences were considered significant at FDR < 0.05. Visualization was conducted using *ggplot2*, Seurat’s plotting tools, and CellChat’s native visualization modules.

### 4.10. Ethics Statement

All datasets analyzed were publicly available and de-identified. No new patient recruitment or experimentation was performed, and therefore institutional review board approval was not required.

## 5. Conclusions

Our study provides a comprehensive single-cell map of the colorectal cancer tumor microenvironment, revealing how distinct epithelial states—particularly CMS2 and CMS3—coordinate immunosuppressive and inflammatory signaling through highly interconnected ligand–receptor networks. By integrating Seurat-based preprocessing, CellChat communication inference, and pathway enrichment analysis, we identified MIF-centered signaling (MIF–CD74–CXCR4/CD44) as a dominant axis linking tumor cells with CD8^+^ T cells, B cells, and SPP1^+^ macrophages, alongside APP–CD74 interactions that are preferentially enriched in CMS3 tumors. These pathways collectively reshape the immune landscape by reinforcing macrophage polarization, impairing cytotoxic lymphocyte function, and sustaining epithelial survival programs. Our findings highlight these communication hubs as promising therapeutic targets, underscoring the potential of modulating MIF-CD74-associated signaling to disrupt immune evasion. Overall, this single-cell analysis deepens our understanding of CRC intercellular communication and provides a mechanistic framework that may inform biomarker development and the design of combination immunotherapies.

## Data Availability

All data were extracted from the Gene Expression Omnibus (GEO) under accession numbers GSE132465 and GSE144735.

## Supplementary Materials

The supporting information can be downloaded at: https://www.mdpi.com/article/10.3390/ijms27031496/s1.

## Abbreviations

The following abbreviations are used in this manuscript:

CRC	Colorectal Cancer
TME	Tumor Microenvironment
scRNA-seq	Single-cell RNA sequencing
PCA	Principal Component Analysis
UMAP	Uniform Manifold Approximation and Projection
CMS1–CMS4	Consensus Molecular Subtypes of CRC
Tregs	Regulatory T cells
Tfh	T follicular helper cells
NK	Natural Killer (cells)
DCs/cDCs	Dendritic Cells/Conventional Dendritic Cells
MIF	Macrophage Migration Inhibitory Factor
APP	Amyloid Precursor Protein
MHC-I	Major Histocompatibility Complex class I
GO	Gene Ontology
KEGG	Kyoto Encyclopedia of Genes and Genomes
DEG	Differentially Expressed Genes
UMIs	Unique Molecular Identifiers
