# Supplementary material for: Single-Cell Mapping Reveals MIF-Centered Immunoregulatory Networks in Colorectal Cancer
Source: Int J Mol Sci. 2026 Feb 3;27(3):1496. doi: 10.3390/ijms27031496 (PMC12897769; doi:10.3390/ijms27031496)
Supplement: Supplementary file 1 [file ijms-27-01496-s001.zip › ijms-4087322-supplementary.pdf]

Supplementary material

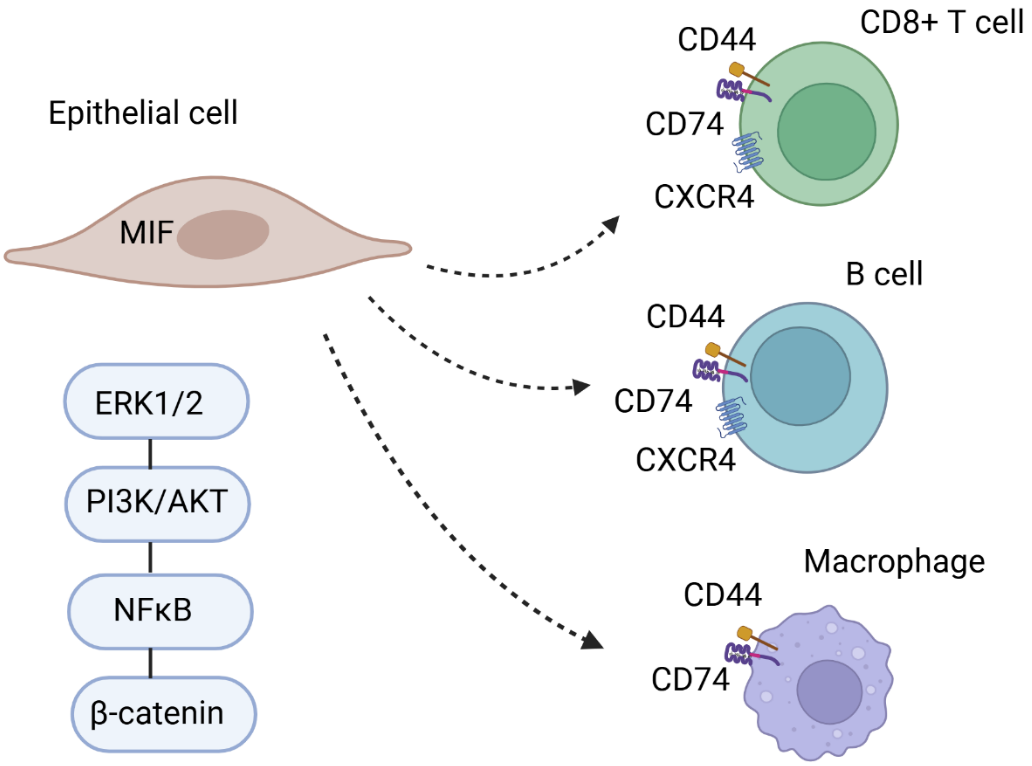

Figure S1. Schematic of MIF–CD74–CD44/CXCR4 signaling across CRC epithelial and immune compartments. Shown are key interfaces relevant to this study. MIF secreted by CRC epithelial cells and immune cells binds CD74 on target cells. CD44 acts as a co-receptor, and CXCR4 can be recruited to form a trimeric complex, amplifying downstream signaling (ERK1/2, PI3K–AKT, NF-κB, β-catenin) that promotes immune cell proliferation, survival, chemotaxis, and immune suppressive differentiation. Shown are key interfaces identified by CellChat in this study: CMS2/3 epithelial cells communicating with $CD8^{+}$ T cells, B cells ($CD19^{+}CD20^{+}$), and $SPP1^{+}$ macrophages via MIF–(CD74+CXCR4) and MIF–(CD74+CD44) axes. Receptor components are annotated on immune cell membranes.

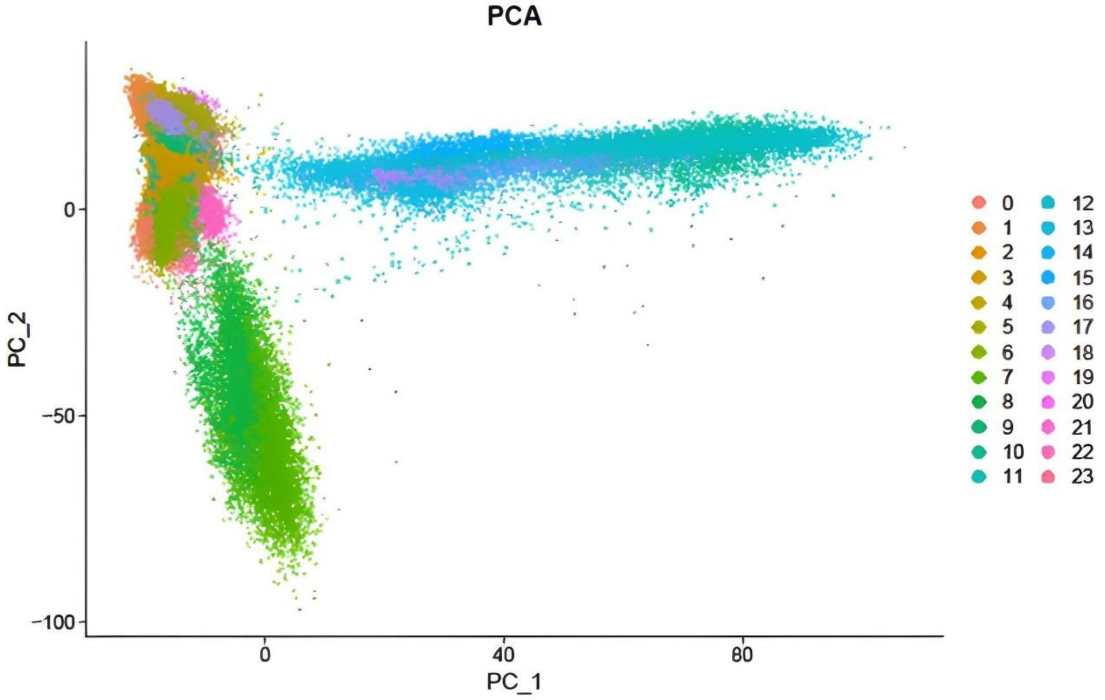

Figure S2. Principal Component Analysis (PCA) plot showing separation of major cell compartments following dataset integration and normalization.
